# Hypokalemia-Induced Rhabdomyolysis Following Prolonged Diarrhea in a Child With Congenital Heart Disease Receiving Loop Diuretics: A Case Report and Literature Review

**DOI:** 10.7759/cureus.104451

**Published:** 2026-02-28

**Authors:** Keiichiro Iwasaki, Mizuki Akiyoshi, Shigeki Nakashima, Kenji Yasuda, Takeshi Taketani

**Affiliations:** 1 Department of Pediatrics, Shimane University Faculty of Medicine, Izumo, JPN

**Keywords:** diarrhea, hypokalemia, loop diuretics, pediatric, rhabdomyolysis

## Abstract

Hypokalemia is a rare but important cause of rhabdomyolysis in children. Unlike typical rhabdomyolysis, hyperkalemia may be absent because the triggering factor is hypokalemia, potentially delaying diagnosis. We report a five-year-old boy with hypoplastic left heart syndrome after a Fontan procedure who developed severe hypokalemia and rhabdomyolysis following prolonged diarrhea while receiving loop diuretics. He presented with acute lower extremity weakness and inability to stand. Laboratory evaluation revealed potassium 1.9 mmol/L and creatine kinase 9,013 U/L, peaking at 32,908 U/L. Urinalysis showed myoglobinuria, and electrocardiography demonstrated ST depression and prominent U waves. Intravenous potassium replacement and diuretic adjustment resulted in rapid clinical recovery without acute kidney injury. A review of previously reported pediatric cases suggests that potassium depletion from gastrointestinal losses or renal wasting predisposes children to rhabdomyolysis. Early recognition and prompt electrolyte correction are essential.

## Introduction

Rhabdomyolysis is characterized by skeletal muscle breakdown and leakage of intracellular components such as creatine kinase (CK), myoglobin, and electrolytes into the circulation. In children, infections, trauma, and metabolic disorders are common causes. Electrolyte disturbances, particularly hypokalemia, are uncommon [1,2]. Rhabdomyolysis may result in life-threatening complications such as acute kidney injury, severe electrolyte disturbances, cardiac arrhythmias, and disseminated intravascular coagulation, underscoring the importance of early recognition and management [1,2].

Potassium is essential for skeletal muscle perfusion and cellular energy metabolism. Severe depletion can impair Na⁺/K⁺-ATPase activity, promote intracellular calcium overload, and lead to ATP exhaustion and myocyte necrosis [2,3]. Importantly, hyperkalemia, commonly expected in rhabdomyolysis, may not occur in hypokalemic cases [4].

This diagnostic challenge is particularly important in children with congenital heart disease receiving loop diuretics, as chronic renal potassium wasting compounded by persistent diarrhea can lead to profound total body potassium depletion and subsequent rhabdomyolysis. Our case highlights this preventable yet underrecognized risk in a vulnerable pediatric population.

We report a child with congenital heart disease receiving loop diuretics who developed hypokalemia-induced rhabdomyolysis following prolonged diarrhea, along with a brief review of the literature.

## Case presentation

A five-year-old boy presented with acute lower extremity weakness and inability to stand. He had a history of hypoplastic left heart syndrome and had undergone a Fontan procedure at four years and 10 months of age. His daily medications included furosemide (1 mg/kg/day), spironolactone (1 mg/kg/day), warfarin, bisoprolol, and flecainide. He also had mild-to-moderate intellectual disability and marked food selectivity, with a limited diet consisting primarily of white rice and meat-based dishes. There was no family history of periodic paralysis

Ten days before admission, he developed profuse watery diarrhea occurring up to 20 times per day, accompanied by poor oral intake. The diarrhea gradually improved; however, on the day of presentation, he developed progressive lower limb weakness and became unable to rise from a sitting position. He was brought to our hospital for evaluation.

On examination, his temperature was 36.9°C, oxygen saturation was 88% on room air (baseline for Fontan physiology), heart rate was 90 beats/min, and blood pressure was 92/59 mmHg. He was hemodynamically stable, and physical examination was unremarkable. Neurologically, he could maintain a sitting posture and move his extremities spontaneously but was unable to stand or bear weight because of muscle weakness.

Laboratory testing revealed severe hypokalemia (1.9 mmol/L) and markedly elevated CK levels (9,013 U/L) (CK-MB 119.3 ng/mL), consistent with skeletal muscle injury. Aspartate aminotransferase (118 U/L) and lactate dehydrogenase (721 U/L) were also elevated. Renal function was preserved (blood urea nitrogen 6.4 mg/dL, creatinine 0.49 mg/dL). C-reactive protein (CRP) was mildly elevated (1.12 mg/dL), likely due to recent gastroenteritis. Urinalysis showed positive occult blood without red blood cells, suggestive of myoglobinuria. Venous blood gas analysis showed no significant metabolic acidosis (Table 1). Virus isolation tests on the patient's stool from diarrhea were negative.

**Table 1:** Laboratory findings on admission RBCs: Red blood cells; WBCs: White blood cells; PCO_2_: Partial pressure of carbon dioxide; HCO_3_^-^: Bicarbonate; AST: Aspartate aminotransferase; ALT: Alanine aminotransferase; LDH: Lactate dehydrogenase; CK: Creatine kinase; CK-MB: Creatine kinase–MB isoenzyme (myocardial band); BUN: Blood urea nitrogen; CRP: C-reactive protein; BNP: Brain natriuretic peptide; TnI: Cardiac troponin I; TSH: Thyroid-stimulating hormone.

Parameters	Value	Unit	Reference range
*Urine*			
Protein	Negative		Negative
Occult blood	2+		Negative
*Urinary sediment*			
RBCs	0–4	High-power field	0–4
WBCs	0–5	High-power field	0–5
*Venous blood gas*			
pH	7.4		7.31–7.41
pCO_2_	43.4	mmHg	41–51
HCO_3_^-^	26.3	mmol/L	22–26
Base excess	1.6	mmol/L	−2 to +2
Glucose	104	mg/dL	70–110
Lactate	3.9	mmol/L	0.5–2.0
*Complete blood count*			
WBC	10,000	/μL	5,000–14,500
Hemoglobin	17.6	g/dL	11.5–13.5
Platelet	344 × 10^3^	/μL	150–350 × 10^3^
*Blood chemistry*			
Total protein	7.1	g/dL	6.5–8.0
Albumin	4.2	g/dL	3.6–5.0
AST	118	U/L	10–40
ALT	19	U/L	5–40
LDH	721	U/L	120–240
CK	9,013	U/L	40–200
CK-MB	119.3	ng/mL	<5
BUN	6.4	mg/dL	8–20
Creatinine	0.49	mg/dL	0.4–1.1
Sodium	142	mmol/L	135–145
Chloride	105	mmol/L	98–108
Potassium	1.9	mmol/L	3.5–5.0
Calcium	9.7	mg/dL	8.6–10.2
Magnesium	2.2	mg/dL	1.7–2.2
CRP	1.12	mg/dL	<0.2
BNP	47.2	pg/mL	<100
TnI	<0.01	ng/mL	<0.01
Free T4	1.4	ng/dL	0.90–1.70
TSH	4.9	mIU/mL	0.61–4.23

**Figure 1:**
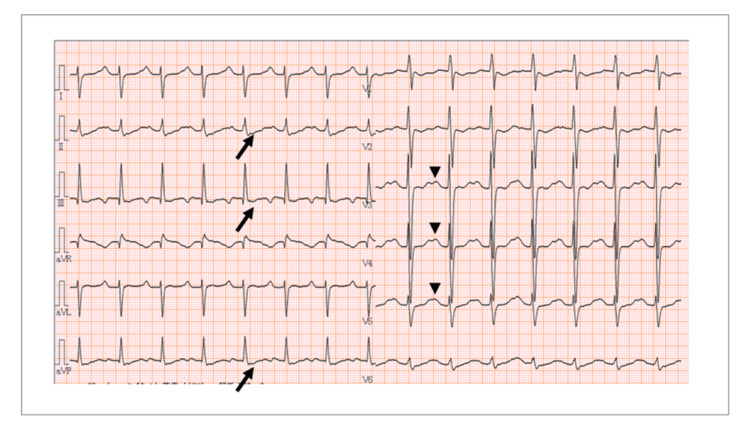
Electrocardiographic findings suggestive of hypokalemia The arrows indicate the ST-segment depression in leads II, III, and aVF, and the arrowheads indicate prominent U waves in leads V3–V5.

Electrocardiography demonstrated ST-segment depression and prominent U waves, findings consistent with hypokalemia (Figure 1).

**Figure 2:**
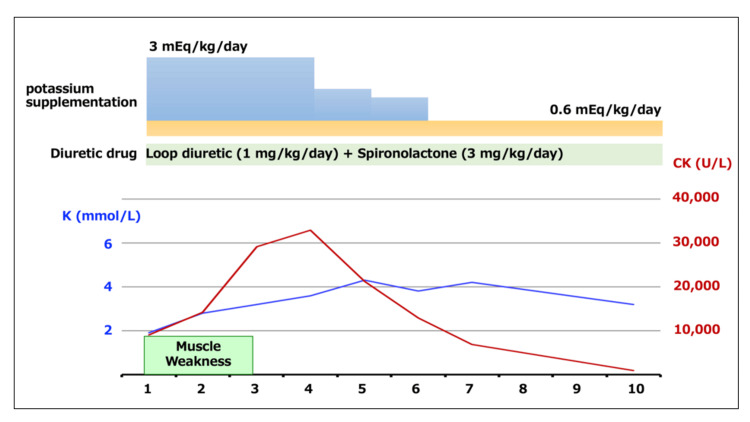
Clinical course

Based on these findings, hypokalemia-induced rhabdomyolysis was diagnosed. Intravenous potassium supplementation was initiated at 0.6-3 mEq/kg/day with continuous cardiac monitoring. The furosemide dose was reduced, and spironolactone was increased. CK levels peaked at 32,908 U/L on hospital day 2 and subsequently declined with treatment. Muscle strength improved gradually, and the patient regained the ability to stand within several days. No acute kidney injury or other complications occurred. He was discharged after normalization of potassium levels and continued clinical improvement (Figure 2). Urine output was maintained throughout the hospitalization period. To date, eight months after the onset of symptoms, no similar symptoms have been observed.

## Discussion

**Figure 3:**
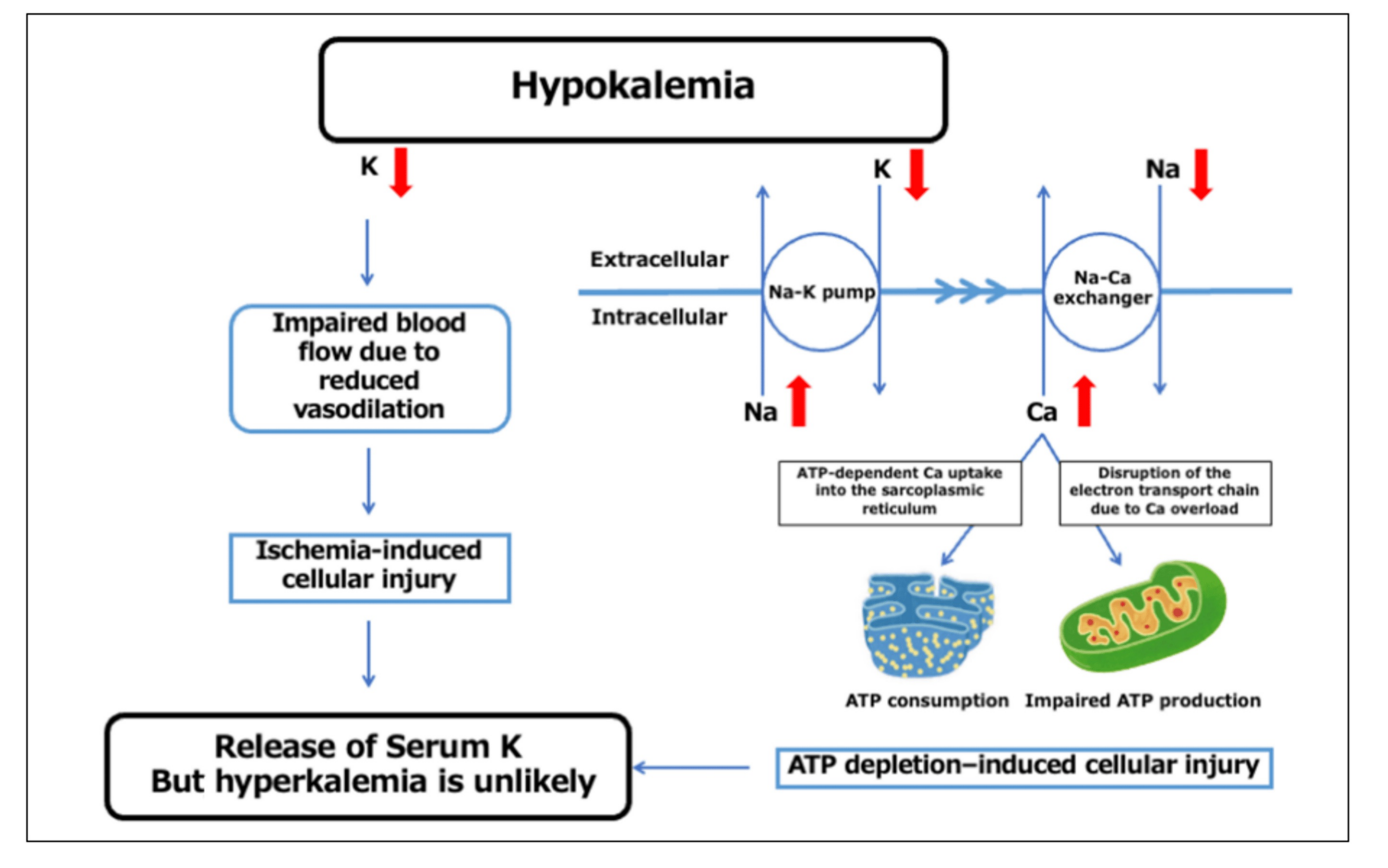
Proposed mechanism of hypokalemia-induced rhabdomyolysis and absence of hyperkalemia Hypokalemia impairs Na⁺/K⁺ pump and Na⁺/Ca²⁺ exchanger activity, leading to intracellular Ca²⁺ overload, mitochondrial dysfunction, ATP depletion, and muscle cell injury (rhabdomyolysis). In typical rhabdomyolysis, potassium release from injured muscle cells causes hyperkalemia. However, under hypokalemic conditions, extracellular potassium remains low, making hyperkalemia unlikely.

Rhabdomyolysis in children is most commonly associated with infection, trauma, or metabolic disorders. Electrolyte abnormalities are less frequently recognized causes. Among these, hypokalemia is a rare but clinically important and potentially reversible trigger. Potassium is essential for skeletal muscle perfusion and cellular energy metabolism; severe depletion disrupts ion homeostasis, causing intracellular calcium overload, ATP depletion, and myocyte necrosis (Figure 3) [2,3,5].

Unlike typical rhabdomyolysis, which often results in hyperkalemia due to potassium release from damaged muscle cells, hypokalemia-associated rhabdomyolysis may not demonstrate elevated serum potassium levels [4]. This paradox can delay diagnosis because clinicians may not suspect muscle injury in the absence of hyperkalemia. Our case highlights this important clinical distinction.

Several risk factors likely contributed to potassium depletion in this patient. First, prolonged diarrhea resulted in substantial gastrointestinal potassium loss. Second, chronic use of loop diuretics promoted ongoing renal potassium wasting. Third, reduced oral intake and selective eating habits due to intellectual disability further limited potassium replenishment. The combination of these factors likely resulted in severe total body potassium depletion sufficient to precipitate muscle injury.

Previous pediatric reports have described hypokalemia-induced rhabdomyolysis in association with renal tubular disorders, antifungal agents, and gastrointestinal potassium loss (Table 2) [6-13]. A review of the literature indicates that most affected children present with muscle weakness or gait disturbance and serum potassium levels below 2.2 mmol/L. Consistent with these reports, our patient developed profound weakness with a potassium level of 1.9 mmol/L and marked CK elevation.

**Table 2:** Clinical features of pediatric rhabdomyolysis caused by hypokalemia y: Year-old; F: Female; M: Male; K: Potassium; CK: Creatine kinase. ^#^ indicates that the underlying disease was diagnosed after the onset of rhabdomyolysis; * indicates that the CK values were expressed as “X fold above upper limit of normal” because of differences in the measurement methods; ^¶^ indicates the present case.

Age/Sex	Underlying disease	Trigger	Symptoms	K (mmol/L)	CK (U/L)	Reference number
1yF	Renal tubular acidosis (RTA) #	Upper respiratory infection (URI)	Muscle weakness	1.4	14-fold*	[8]
3yM	Autism spectrum disorder	Food selectivity	Gait disturbance	1.98	4,705	[12]
4yF	Bartter syndrome	Enteritis, drug discontinuation	Muscle weakness	<1.5	11-fold*	[8]
4yF	Bartter syndrome	Chronic hypokalemia	None	1.9	1,680	[7]
7yM	RTA	URI, drug discontinuation	Muscle weakness	1.9	18-fold*	[8]
9yF	Fungal infection	Voriconazole	Muscle weakness, gait disturbance	2.2	13,006	[5]
10M	Fungal infection	Amphotericin B	Muscle weakness, muscle pain, gait disturbance	1.7	3,937	[11]
10yM	11β-hydroxylase deficiency	Drug discontinuation	Muscle weakness, gait disturbance	1.4	8,100	[9]
13yF	Gitelman syndrome	Chronic hypokalemia	Muscle weakness, muscle pain	2.1	1,248	[10]
14yM	ICOS deficiency	Chronic diarrhea	Muscle weakness, muscle pain	1.9	1,979	[6]
15yM	Gitelman syndrome^#^	Unknown	Muscle weakness, muscle cramp	2.1	7.7-fold*	[8]
4yM^¶^	Congenital heart disease	Food selectivity, enteritis, furosemide	Muscle weakness, gait disturbance	1.9	9,013	This case

Early recognition and prompt correction of hypokalemia are essential to prevent complications such as acute kidney injury. In our patient, aggressive potassium replacement and diuretic adjustment resulted in rapid clinical and biochemical recovery without renal impairment. This favorable outcome underscores the importance of timely electrolyte evaluation in children presenting with acute muscle weakness, particularly those with known potassium-loss risk factors. Many patients with rhabdomyolysis accompanied by hypokalemia present with chronic hypokalemia due to underlying diseases or medications; therefore, hypokalemia may be merely an association rather than a causal relationship. However, given that symptoms and laboratory values in many of these cases improved rapidly with potassium supplementation, and considering the mechanism described in Figure 3, we inferred that hypokalemia triggered the onset of rhabdomyolysis in this case.

This case has several limitations. As this is a single case report, the findings may not be generalizable. As a single observation, causality cannot be definitively established, and spontaneous recovery cannot be completely excluded. Nevertheless, the temporal association between potassium correction and rapid improvement strongly supports hypokalemia as the primary mechanism. Further accumulation of similar cases is needed to better define the incidence and risk factors of hypokalemia-associated rhabdomyolysis in children.

## Conclusions

Hypokalemia should be considered in the differential diagnosis of pediatric rhabdomyolysis, especially in patients with diarrhea, use of loop diuretics, or chronic medical conditions predisposing to potassium loss. Routine electrolyte monitoring and early intervention may prevent severe muscle injury and renal complications.
